# FACILITATE: A real-world, multicenter, prospective study investigating the utility of a rapid, fully automated real-time PCR assay versus local reference methods for detecting epidermal growth factor receptor variants in NSCLC

**DOI:** 10.3389/pore.2023.1610707

**Published:** 2023-01-31

**Authors:** Anke Behnke, Anne Cayre, Giovanna De Maglio, Giuseppe Giannini, Lionel Habran, Marina Tarsitano, Massimiliano Chetta, David Cappellen, Alexandra Lespagnol, Cecile Le Naoures, Gabriella Massazza, Annarita Destro, Irina Bonzheim, Achim Rau, Achim Battmann, Bettina Kah, Emmanuel Watkin, Michael Hummel

**Affiliations:** ^1^ Charité-Universitätsmedizin Berlin, Institute of Pathology and Berlin Institute of Health, Corporate Member of Freie Universität Berlin and Humboldt-Universität zu Berlin, Berlin, Germany; ^2^ Département de Pathologie, Centre Jean-Perrin, Clermont-Ferrand, France; ^3^ Azienda Sanitaria Universitaria Friuli Centrale, Pathology Department, Santa Maria della Misericordia Hospital, Udine, Italy; ^4^ Department Molecular Medicine, Università di Roma La Sapienza, Rome, Italy; ^5^ Anatomopathology Department, CHU Liège, Liège, Belgium; ^6^ Di Laboratorio, A.O.R.N. Cardarelli, Medical Genetics Laboratory, and Ospedale Antonio Cardarelli, U.O.C. di Genetica Medica, Naples, Italy; ^7^ Service de Biologie des Tumeurs, Centre Hospitalier Universitaire de Bordeaux, Hôpital du Haut Lévêque, Pessac, France; ^8^ CHU de Rennes, Laboratoire de Génétique Somatique des Cancers, Rennes, France; ^9^ CHU de Rennes, Service d’Anatomie et Cytologie Pathologiques, Rennes, France; ^10^ Dipartimento Medicina di Laboratorio Anatomia Patologica, ASST Papa Giovanni XXIII, Bergamo, BG, Italy; ^11^ Pathology Department, Humanitas Clinical and Research Center—IRCCS, Milan, Italy; ^12^ Institute of Pathology and Neuropathology, Eberhard Karls University of Tübingen and Comprehensive Cancer Center, University Hospital Tübingen, Tübingen, Germany; ^13^ Institut für Pathologie und Zytodiagnostik am Krankenhaus Nordwest, Frankfurt, Germany; ^14^ Institut für Hämatopathologie Hamburg, Hamburg, Germany; ^15^ CYPATH, Villeurbanne, France

**Keywords:** non-small cell lung cancer, epidermal growth factor receptor, DNA mutational analysis, clinical decision-making, turnaround time

## Abstract

Accurate testing for epidermal growth factor receptor (*EGFR*) variants is essential for informing treatment decisions in non-small cell lung cancer (NSCLC). Automated diagnostic workflows may allow more streamlined initiation of targeted treatments, where appropriate, while comprehensive variant analysis is ongoing. FACILITATE, a real-world, prospective, multicenter, European study, evaluated performance and analytical turnaround time of the Idylla™ EGFR Mutation Test compared with local reference methods. Sixteen sites obtained formalin-fixed paraffin-embedded biopsy samples with ≥ 10% neoplastic cells from patients with NSCLC. Consecutive 5 μm sections from patient samples were tested for clinically relevant NSCLC-associated *EGFR* variants using the Idylla™ EGFR Mutation Test and local reference methods; performance (concordance) and analytical turnaround time were compared. Between January 2019 and November 2020, 1,474 parallel analyses were conducted. Overall percentage agreement was 97.7% [*n* = 1,418; 95% confidence interval (CI): 96.8–98.3], positive agreement, 87.4% (*n* = 182; 95% CI: 81.8–91.4) and negative agreement, 99.2% (*n* = 1,236; 95% CI: 98.5–99.6). There were 38 (2.6%) discordant cases. Ninety percent of results were returned with an analytical turnaround time of within 1 week using the Idylla™ EGFR Mutation Test versus ∼22 days using reference methods. The Idylla™ EGFR Mutation Test performed well versus local methods and had shorter analytical turnaround time. The Idylla™ EGFR Mutation Test can thus support application of personalized medicine in NSCLC.

## Introduction

Molecular profiling in non-small cell lung cancer (NSCLC) is of key importance in identifying presence of oncogenic drivers, allowing implementation of targeted treatment approaches where applicable [1]. The epidermal growth factor receptor (*EGFR*) is an established oncogenic target in NSCLC [2–4]. A meta-analysis of 456 studies showed that *EGFR* variants have a prevalence of 17.4% [95% confidence interval (CI): 15.8–18.9] and 38.8% (95% CI: 36.8–40.8) in Caucasian and Asian patients with NSCLC, respectively [5]. The 25 currently described exon 19 deletion variants, which are referred to as exon 19 deletions (Ex19del) and the c.2573T>G [p.Leu858Arg (commonly referred to as L858R)] variant are the most common and represent approximately 90% of all *EGFR* pathogenic variants [6].

EGFR tyrosine kinase inhibitors (TKIs) are used in the treatment of NSCLC harboring EGFR-TKI sensitizing pathogenic variants such as Ex19del and p.Leu858Arg [7–10]. Resistance to EGFR-TKIs can be acquired, which depending on the specific EGFR-TKI used, can involve other *EGFR* pathogenic variants such as c.2369C>T [p.Thr790Met (commonly referred to as T790M)] and c.2389T>A [p.Cys797Ser (commonly referred to as C797S)] [11]. It should be noted that tumor cells in which the p.Cys797Ser and p.Thr790Met variants occur together in the cis-configuration (on the same allele) were shown to be insensitive to currently approved EGFR-TKIs, including third-generation EGFR-TKIs [12]. Cases in which the *EGFR* p.Cys797Ser and p.Thr790Met variants occur together in the trans-configuration (on separate alleles) may be sensitive to first- and third-generation EGFR-TKI-combination treatment [12]. Currently, the preferred first-line treatment in patients with EGFR-TKI-sensitizing variant-positive (e.g., Ex19del, p.Leu858Arg) advanced NSCLC is osimertinib [4, 13]. Osimertinib is a third-generation, irreversible, oral EGFR-TKI that potently and selectively inhibits EGFR-TKI-sensitizing and *EGFR* p.Thr790Met variants with demonstrated efficacy in *EGFR* variant-positive NSCLC, including in patients with central nervous system (CNS) metastases [10,14–18].

Guidelines, endorsements and recommendations concerning advanced NSCLC from the European Society for Medical Oncology (ESMO) and American Society of Clinical Oncology, among other societies, suggest that patients should be tested for oncogenic drivers at the time of diagnosis due to the benefits of targeted treatment [4,19–21]. Per ESMO guidelines, *EGFR* profiling must cover Ex19del variants and p.Leu858Arg variants in exon 21; however, complete coverage of genomic alterations across exons 18–21 is recommended [4]. While the *EGFR* p.Thr790Met exon 20 substitution variant is rarely found in patients with treatment-naïve *EGFR* mutated NSCLC, germline p.Thr790Met has been reported [4, 22]. Implications of this variant in patients with previously untreated disease are unclear, but with the availability of osimertinib, p.Thr790Met testing on relapse with earlier-generation EGFR-TKIs is mandatory [4].

Clinical testing for *EGFR* variants may involve single variant-genotyping technologies, but multiplex gene-sequencing technologies such as next-generation sequencing (NGS) have been adopted as the standard approach in many instances [4, 23]. These latter technologies are used with targeted gene panels and can screen several genes simultaneously [24–26].

Guidelines specify that molecular testing results for recommended predictive biomarkers should be available 10 days after tissue acquisition [27]. Despite this, lengthy analytical turnaround times (aTAT) remain an important potential barrier to provision of genomic data ahead of treatment initiation. In the UK for example, the National Cancer Audit found that irrespective of the test performed, the median turnaround time was 18 days [27]. Based on clinical experience regarding test type, comprehensive testing such as with NGS (currently the most common approach) is generally associated with longer turnaround times compared with simpler techniques based on polymerase chain reaction (PCR); 5–14 days (or more) versus 1–7 days, respectively [28, 29]. Moreover, while many large academic hospitals have inhouse testing facilities suitable for performing comprehensive variant screening, others must send samples to external laboratories; this can risk additional delays [30].

Lengthy turnaround times can result in patients and clinicians feeling it is necessary to choose between waiting for variant test results and starting treatment plans based on incomplete information, potentially resulting in initiation of sub-optimal therapies [28, 31]. This is significant in frail patients, patients with high symptom burden, and those with advanced NSCLC (e.g., stage IV) who can deteriorate rapidly while waiting for test results, as flagged in several reports [28, 32, 33]. Prompt molecular test turnaround times are also important in the stage I‒III resectable NSCLC setting. Unlike in the advanced setting, few targeted treatment strategies are available (which require prior molecular testing) in resectable NSCLC. *EGFR* is one such target in this setting that is appliable for targeted treatment. This follows the approval of adjuvant osimertinib based on results of the ADAURA study [34]. In line with this, variant testing in resectable tissue is now recommended per the ESMO guidelines [35].

Limited availability of tissue samples of sufficient size and sub-optimal workflows can pose additional barriers to pre-treatment *EGFR* variant testing. This can put restrictions on clinical laboratories regarding number and types of tests possible [31, 36, 37]. Methods and streamlined workflows that permit accurate *EGFR* variant testing, shorten turnaround time and make best use of samples are imperative to improve care in NSCLC [38]. This is important in relation to the availability of treatment strategies such as EGFR-TKIs, which can target various *EGFR* variants.

The Idylla™ EGFR Mutation Test performed on the Biocartis Idylla™ System (Biocartis, Belgium), is a fully automated real-time PCR (RT-PCR)-based test designed specifically to detect common clinically relevant NSCLC-associated EGFR variants. It can be used directly on formalin-fixed and paraffin-embedded (FFPE) tissue sections with neoplastic cell content of at least 10%. This *EGFR* specific test has been evaluated for clinical utility in lung cancer workflows. Previous studies demonstrated its benefits in facilitating rapid and accurate testing [39]. For instance, it has been examined regarding *EGFR* variant screening [40] and reflex testing [29]. In these studies, turnaround times ranged from 1 to 3 days [29, 40] and concordance with NGS was approximately 98.5% [40]. Recently, a single center-experience study described use of the Idylla™ EGFR Mutation Test, with NGS in a sequential multi-test approach, and reported an average turnaround time of 2 days and 96.4% concordance with NGS [41]. In addition, the Idylla™ EGFR Mutation Test requires modest input samples quantities, making efficient use of samples [42].

To help improve *EGFR* variant clinical testing and examine use of rapid variant testing methods in NSCLC, the FACILITATE (fast, accurate, Idylla™-based investigation of turnaround time in *EGFR* testing) study was performed. FACILITATE was a real-world, multicenter prospective European study evaluating performance and turnaround time of the Idylla™ EGFR Mutation Test compared with reference methods in real-life NSCLC settings.

## Materials and methods

### Patient criteria

For inclusion in this study, samples must have been obtained as part of routine clinical practice from patients with confirmed NSCLC (any stage); and sample *EGFR* variant status must have been unknown prior to *EGFR* variant analysis with the Idylla™ EGFR Mutation Test. No further inclusion and exclusion criteria were included in the study design as one of the aims was to conduct the study under real-life conditions, including the diversity of incoming NSCLC samples, in order to be representative of everyday clinical practice.

### Study design

**FIGURE 1:**
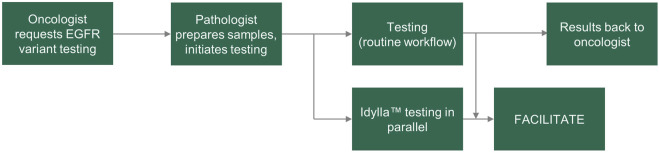
FACILITATE study workflow. *EGFR*, epidermal growth factor receptor.

FACILITATE was a real-world, multicenter, prospective study across 16 clinical sites in Belgium, France, Germany and Italy. Samples were obtained prospectively from patients and prepared as 5 μm FFPE human tumor samples. Consecutive sections were tested for *EGFR* variants in parallel at each site using the Idylla™ EGFR Mutation Test and a local reference method (Figure 1). Sample sections used in the Idylla™ EGFR Mutation Test were required to have a neoplastic cell content of at least 10%; if sections had less than 10% neoplastic cell content, macro-dissection was performed. Sample sections used in each of the local reference methods must have fulfilled the sample requirements as noted in respective manufacturer’s instructions for use. Time points for the following were recorded in each case: sample receipt by laboratory and results ready to be sent to the clinician.

### Ethics statement

Use of patient samples in this study was approved by the respective local ethics committees and was in accordance with the Declaration of Helsinki.

### Objectives

The main objectives were to assess utility of the Idylla™ EGFR Mutation Test in the European clinical setting using real-life patient NSCLC samples and to assess its impact on aTAT in accurate reporting of *EGFR* results. aTAT was defined as time between laboratory receipt of sample and when the molecular diagnostic test result was ready for the clinician. Performance was also assessed across centers and described by the level of concordance between the Idylla™ EGFR Mutation Test and the local reference method.

### Idylla™ EGFR mutation test

The Idylla™ EGFR Mutation Test performed on the Biocartis Idylla™ System (Biocartis, Belgium) is an *in vitro* sample-to-result diagnostic test with a rapid technical run time (approximately 150 min) for the qualitative detection of 51 common NSCLC-associated *EGFR* variants across exons 18–21 [43]. The limit of detection in terms of allele frequency (AF) is 5%. All 51 *EGFR* variants detectable with the Idylla™ EGFR Mutation Test are presented in Supplementary Table S1 [43].

During testing, disposable Idylla™ EGFR Mutation Test cartridges were loaded with FFPE human NSCLC tissue sections without prior manual deparaffinization or FFPE pre-processing. Cartridges were inserted into the Idylla™ instrument, per the manufacturer’s instructions. The Idylla™ console and instruments were Conformité Européene marked. Inside the Idylla™ cartridge, deoxyribonucleic acid (DNA) was liberated from FFPE material using a combination of reagents, enzymes, heat and high intensity-focused ultrasound. Within the cartridge, allele-specific multiplex PCR was performed for the amplification of specific mutated *EGFR* gene sequences. Conserved regions of the *EGFR* genes served as sample processing controls and as a measure of the amplifiable DNA in each sample, which is represented in each case by the quantitation cycle (Cq) value. The conserved regions were amplified in parallel with mutated *EGFR* gene sequences, where present. All required consumables were provided in the cartridge. The official *in vitro* diagnostic-certified Idylla™ console report was used as the final Idylla™ result.

The Idylla™ Explore tool was used to facilitate discordance analysis and invalid or non-processable result identification and investigation. The Idylla™ Explore tool is available to registered users *via* a web browser and allowed users to view the raw amplification data in more detail. This Idylla™ Explore tool serves as data visualization aid for research purposes only.

### Reference methods

Local reference methods used in parallel with the Idylla™ EGFR Mutation Test across sites in FACILITATE, included amplicon- or hybrid capture-based NGS, cobas® EGFR Test, Sanger sequencing, Therascreen® EGFR Rotor-Gene Q (RGQ) PCR, MassARRAY® and Entrogen RT-PCR (see Supplementary Table S2 for more details).

### Discordance analyses

After the results of the in-parallel run Idylla™ EGFR Mutation Test and reference methods were available for a sample, additional steps were taken, and where relevant, third method analysis was conducted. The third method selected was dependent on local availability; it could not be the Idylla™ EGFR Mutation Test or the reference method used at the respective site. To identify, verify, and if possible, understand or resolve instances of discordance or invalid or non-processable results, in-depth curve investigation was performed by the Idylla™ EGFR Mutation Test manufacturer. If needed, digital droplet PCR (ddPCR) was performed on DNA retrieved from the Idylla™ EGFR Mutation Test cartridge or relevant reference method equipment. Additionally, or alternatively, appropriate FFPE tissue sections and/or DNA retrieved from the Idylla™ cartridge were analyzed using a BioRad (Hercules, CA) QX100 system per the manufacturer’s instructions.

Cases in which the Idylla™ EGFR Mutation Test detected an *EGFR* variant but the reference method did not, were defined as “discordant positive”; cases in which the contrary occurred were defined as “discordant negative.” Cases in which samples were concordant for the primary variant but discordant for a secondary variant were also identified. Cases in which *EGFR* variants were detected by the reference method but not by the Idylla™ EGFR Mutation Test because the specific variant sequences were not part of the Idylla™ 51-member panel, were defined as “discordant by design.”

### Statistical methods

Agreement between the Idylla™ EGFR Mutation Test and the comparator method was evaluated based on point estimates for overall, positive and negative percentage diagnostic agreement (OPA, PPA and NPA, respectively) together with 95% two-sided Wilson score CIs. Per 2 × 2 concordance table agreement calculations with 95% CIs including lower and upper limits, it was determined that a minimum of approximately 700 parallel analyses would be required to obtain a lower limit ≥ 90% (assuming a prevalence of 15% and a concordance of 95%). However, to account for invalid or non-processable samples or dropouts, we aimed to conduct 1,500 analyses. aTAT is reported as median [Q1–Q3 (Quartile 1–Quartile 3)] days from time of laboratory receipt of sample to availability of molecular diagnostic test result to the clinician.

## Results

### Patient demographics and samples

Between January 2019 and November 2020, 1,474 patients with NSCLC (any stage) were included in 16 sites across France (five sites), Germany (five sites), Italy (five sites) and Belgium (one site), with each site processing approximately 100 paired samples for prospective *EGFR* variant analysis. Detailed patient demographics were not collected for analysis.

**FIGURE 2:**
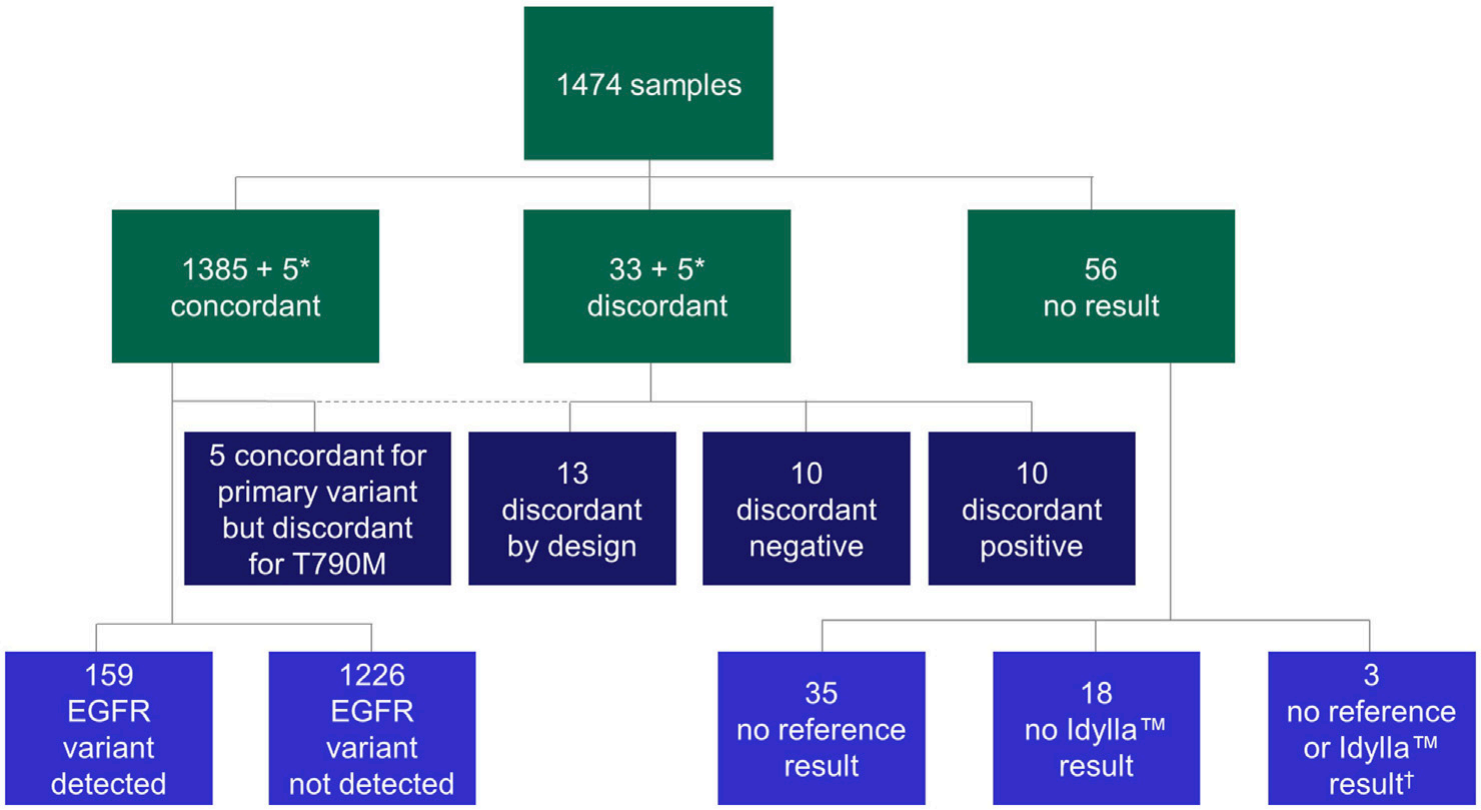
Flow chart of samples. In all cases, concordance was calculated on primary variant level (variant vs. variant). Discordant positive: variant by Idylla™ EGFR Mutation Test, no variant by reference method; discordant negative: variant by reference method, no variant by Idylla™ EGFR Mutation Test; discordant by design: rare *EGFR* variant not in panel of Idylla™ EGFR Mutation Test. *A total of five samples were concordant for the primary variant but discordant for secondary variant; therefore, the same five samples appear in both the concordant and discordant categories. ^†^No results were available from the respective reference tests or the Idylla™ EGFR Mutation Test for these three samples. *EGFR*, epidermal growth factor receptor.

Using these samples, a total of 1,474 parallel analyses (Idylla™ EGFR Mutation Test vs. the local reference methods) were conducted to determine *EGFR* variant status (Table 1). The following local reference methods [*n* samples analyzed (% of total analyses) over *n* sites at which the respective method was employed] were used: Amplicon-based NGS [*n* = 865 (58.7%) over 10 sites], hybrid capture NGS and cobas® [*n* = 83 (5.6%) and *n* = 20 (1.4%), respectively, both performed at one site], Sanger sequencing [*n* = 100 (6.8%) in one site], Therascreen® EGFR RGQ PCR [*n* = 101 (6.9%) in one site], MassARRAY® [*n* = 200 (13.6%) over two sites] and Entrogen RT-PCR [*n* = 105 (7.1%) in one site]. An overview of the sample flow is provided in Figure 2. Results of pre-analytical investigations, including sample neoplastic cell content are also provided in Table 1.

**TABLE 1:** Frequency of *EGFR* variants detected, pre-analytical and analytical details per site.

Site	Reference method used	No. of analyses	Frequency of sections with *EGFR* variants (%)	Testing site	Neoplastic cells (mean %, ±SD)	*EGFR* control Cq values per site (mean cycles, ±SD)
1	NGS^a^	100	10	Comprehensive cancer center	63 ± 23	23.0 ± 3.10
2	NGS^b^	110	14	Private lab	ND	21.3 ± 2.09
3	NGS^a^	101	16	Academic public hospital	ND	22.6 ± 2.88
4	NGS^a^	14	ND	Community public hospital	ND	21.6 ± 3.01
5	HC or cobas®^a^	103	12	Private lab	49 ± 23	23.2 ± 2.52
6	NGS^a^	109	6	Academic public hospital	58 ± 22	22.4 ± 2.39
7	NGS^a^	31	ND	Community public hospital	62 ± 19	23.9 ± 2.14
8	MassARRAY®^a^	102	18	Academic public hospital	68 ± 20	23.9 ± 2.59
9	Entrogen RT-PCR^a^	105	12	Community public hospital	68 ± 22	22.2 ± 2.56
10	Therascreen® EGFR RGQ PCR^a^	101	10	Community public hospital	52 ± 22	21.6 ± 2.43
11	MassARRAY®^a^	98	16	Community public hospital	ND	24.0 ± 1.20
12	Sanger sequencing^a^	100	13	Community public hospital	56 ± 27	22.5 ± 2.50
13	NGS^b^	197	5	Academic public hospital	45 ± 26	22.4 ± 2.97
14	NGS^a^	99	15	Academic public hospital	ND	23.7 ± 2.79
15	NGS^a^	54	9	Academic public hospital	65 ± 24	22.1 ± 2.34
16	NGS^a^	50	12	Academic public hospital	45 ± 17	21.2 ± 2.28

^a^ In-house testing.

^b^ External testing.

Data for each reference method (HC-NGS and cobas®) at Site 5 were assessed as one data set due low sample numbers for each method if analyzed individually. Cq, quantification cycle; EGFR, epidermal growth factor receptor; HC, hybrid capture; ND, no data; NGS, next-generation sequencing; RGQ, rotor gene Q; RT-PCR, real-time polymerase chain reaction; SD, standard deviation.

### Mutational analysis

#### Overview

Across the study, 170 (11.5%) and 186 (12.6%) samples tested positive for *EGFR* variants in the Idylla™ EGFR Mutation Test and local reference methods, respectively. The median (range) percentage of analyses in which *EGFR* variants were detected (Table 1) and the OPA (Table 2) across sites were 12.0% (5.0–18.0) and 98.0% (93.5–100.0), respectively. *EGFR* control Cq values were consistent across all 16 sites (Table 1). A summary of the Idylla™ EGFR Mutation Test results versus reference methods results is provided in Table 3.

**TABLE 2:** Performance of the Idylla™ EGFR Mutation Test compared with reference methods across sites.

Site	Reference method	*N* OPA	OPA (%)	*N* aTAT (Δ)^c^	aTAT using references method (days; median Q1–Q3)	aTAT using Idylla™ EGFR mutation test (days; median Q1–Q3)	ΔaTAT (days; median ± Q1-Q3)
1	NGS^a^	99	99.4	98	11.0 (8.0–13.0)	2.0 (1.0–4.0)	8.0 (6.0–11.0)
2	NGS^b^	105	100.0	110	12.5 (9.0–21.0)	1.0 (0.0–2.0)	11.0 (7.0–19.0)
3	NGS^a^	91	97.8	ND	ND	ND	ND
4	NGS^a^	13	100.0	ND	ND	ND	ND
5	HC or cobas®^a^	97	100.0	97	13.0 (12.0–15.0)	2.0 (1.0–4.0)	11.0 (8.0–13.0)
6	NGS^a^	108	99.1	109	6.0 (5.0–8.0)	4.0 (2.0–5.0)	2.0 (1.0–4.0)
7	NGS^a^	31	93.5	ND	ND	ND	ND
8	MassARRAY®^a^	99	100.0	95	4.0 (2.0–5.0)	2.0 (1.0–5.0)	1.0 (0.0–2.0)
9	Entrogen RT-PCR^a^	104	94.2	ND	ND	ND	ND
10	Therascreen® EGFR RGQ PCR^a^	96	100.0	ND	ND	ND	ND
11	MassARRAY®^a^	97	100.0	ND	ND	ND	ND
12	Sanger sequencing^a^	95	95.8	93	6.0 (6.0–7.0)	2.0 (1.0–3.0)	4.0 (4.0–5.0)
13	NGS^b^	170	99.4	164	21.0 (18.8–26.0)	6.0 (3.0–10.5)	14.0 (13.0–16.0)
14	NGS^b^	94	98.9	ND	ND	ND	ND
15	NGS^a^	53	96.2	49	10.0 (7.0–11.0)	1.0 (1.0–2.0)	7.0 (6.0–10.0)
16	NGS^a^	50	100.0	50	6.0 (4.0–8.0)	1.0 (0.0–1.0)	5.0 (2.0–7.0)

^a^ In-house testing.

^b^ External testing.

^c^ Seven sites with no aTAT data for the Idylla™ EGFR Mutation Test had not completed aTAT analyses at the time of data collection or did not record any aTAT data.

∆aTAT, aTAT for reference method—aTAT for Idylla™ EGFR mutation test.

aTAT, analytical turnaround time; EGFR, epidermal growth factor receptor; HC, hybrid capture; NGS, next-generation sequencing; N aTAT(Δ), number of samples that had an aTAT result using both methods; ND, not determined; N OPA, number of samples that had a valid result using both tests for concordance analysis, excluding discordant-by-design cases; OPA (%), overall percentage diagnostic agreement (excluding discordant by design); RGQ, rotor gene Q; RT-PCR, real-time polymerase chain reaction; Q1, Quartile 1 (25%); Q3, Quartile 3 (75%).

**TABLE 3:** Contingency table for Idylla^™^ EGFR Mutation Test and reference methods.

	Reference method				
	All samples				
Idylla^TM^ EGFR mutation test		*EGFR* variant	No *EGFR* variant	No result^a^	Total
	*EGFR* variant	159	10	1	170
	No *EGFR* variant	23	1,226	34	1,283
	No result^a^	4	14	3	21
	Total	186	1,250	38	1,474
	Summary				
	OPA^b^: 97.7% (*n* = 1,418; 95% CI: 96.8–98.3)				
	Positive agreement: 87.4% (*n* = 182; 95% CI: 81.8–91.4)				
	Negative agreement: 99.2% (*n* = 1,236; 95% CI: 98.5–99.6)				
	Excluding 13 discordant-by-design samples				
	*EGFR* variant	159	10	1	170
	No *EGFR* variant	10	1,226	34	1,270
	No result^a^	4	14	3	21
	Total	173	1,250	38	1,461
	Summary				
	OPA^b^: 98.6% (*n* = 1,405; 95% CI: 97.8–99.1)				
	Positive agreement: 94.1% (*n* = 169; 95% CI: 89.5–95.7)				
	Negative agreement: 99.2% (*n* = 1,236; 95% CI: 98.5–99.6)				

^a^ “No result” includes invalid results, error results or not tested results.

^b^ Cases of “no results” were not included.

CI, confidence interval; EGFR, epidermal growth factor receptor; OPA, overall percentage diagnostic agreement.

#### OPA and concordance

As summarized in Table 3, OPA for *EGFR* variant detection comparing the Idylla™ EGFR Mutation Test and the reference methods was 97.7% (*n* = 1,418; 95% CI: 96.8–98.3), with a PPA of 87.4% (*n* = 182; 95% CI: 81.8–91.4) and an NPA of 99.2% (*n* = 1,236; 95% CI: 98.5–99.6). When excluding cases in which *EGFR* variants were detected by the reference method but not by the Idylla™ EGFR Mutation Test because the specific sequences were not part of the Idylla™ 51-member panel (discordant-by-design cases), OPA was 98.6% (*n* = 1,405; 95% CI: 97.8–99.1), with a PPA of 94.1% (*n* = 169; 95% CI: 89.5–95.7) and an NPA of 99.2% (*n* = 1,236; 95% CI: 98.5–99.6). The negative agreement value remained the same as only the positive agreement value is affected by exclusion of discordance-by-design cases. There was no difference in the OPA for all reference methods compared with OPA for NGS method alone, irrespective of inclusion of cases in which there was discordance by design. The 56 cases of “No results” (defined as invalid results, error results or no results because the sample was not tested) analyses were not included in the OPA analysis. The mean [± standard deviation (SD)] quantification Cq for total analyses (*n* = 1,453; invalid or non-processable results cases are not included), concordant analyses (*n* = 1,380) and discordant (+p.Thr790Met) analyses (*n* = 38), were 22.6 (±2.7), 22.6 (±2.7) and 23.6 (±3.0), respectively.

#### Discordance

Overall, 38 (2.6%) cases were discordant across the study. Of these, 10 (26.3%) were discordant positive, 10 (26.3%) were discordant negative, five (13.2%) were discordant for secondary p.Thr790Met but concordant for primary variant and 13 (34.2%) were discordant by design (Figure 2; Supplementary Table S3).

#### Discordant-positive cases

The 10 cases of discordant positive analyses (Idylla™ EGFR Mutation Test, positive; local reference method, negative) included detection of four Ex19del, two exon 20 insertions (Ex20ins), two p.Leu858Arg variants, one p.Ser768Ile variant and one p.Thr790Met variant (Figure 2; Supplementary Table S3). In nine of these 10 cases, third method analysis (i.e., using another available testing method, independent of the Idylla™ EGFR Mutation Test and the respective routine reference method) was performed to investigate the result (Supplementary Table S3). The Idylla™ EGFR Mutation Test result (variant detected) or reference method (variant not detected) result was confirmed in three out of nine and five out of the nine cases, respectively. In one case, in which third method analysis was performed, left-over material in the cartridge was tested with ddPCR, but the results were inconclusive. In the remaining one case, in which third method analysis was not appliable, left-over material in the cartridge could not be tested. The 10 discordant positive cases had a mean total *EGFR* Cq value of 24.2 ± 1.6 cycles.

#### Discordant-negative cases

The 10 cases of discordant-negative analyses (Idylla™ EGFR Mutation Test negative; local reference method positive) included detection of three p.Leu858Arg variants, three p.Leu861Gln variants, one p.Gly719Ala variant, one Ex19del (p.Ser752_Ile759del) variant, one Ex20ins (p.Asp770_Asn771insGly) variant and one case of concurrent p.Gly719X and p.Thr790Met variants (Figure 2; Supplementary Table S3). The p.Gly719X alteration was reported using colloquially used nomenclature that currently identifies three different variants: (i) c.2156G>C (p.Gly719Ala), (ii) c.2155G>A (p.Gly719Ser) and (iii) c.2155G>T (p.Gly719Cys).

In one of the three cases in which the p.Leu858Arg variant was detected, a hematoxylin and eosin-stained FFPE tissue section was used in the Idylla™ cartridge. In the two other cases, NGS reference methods detected p.Leu858Arg and p.Leu861Gln variants, however, the AF was 3% and 4%, respectively. *EGFR* total Cq values in these two cases were 27.3 and 24.6 cycles, respectively.

Third method analysis using ddPCR was performed in two of the overall 10 discordant-negative cases; left-over material in the cartridges was used for third method analyses in both cases. In one of these two cases, ddPCR [screening for p.Gly719Ala, p.Gly719Cys, p.Gly719Cys(2), p.Gly719Ser and p.Thr790Met variants] confirmed that p.Gly719X and p.Thr790Met variations were not present in the sample tested with Idylla™ EGFR Mutation Test. In the other, ddPCR confirmed that p.Leu861Gln was present in the sample tested with the Idylla™ EGFR Mutation Test but with an AF of 0.4%.

For the remaining five discordant-negative cases, no third method analysis was conducted, however, in-depth curve investigations were completed by the Idylla™ EGFR Mutation Test manufacturer. In two of these five cases (one in which p.Leu861Gln was detected and one in which p.Leu858Arg was detected), an amplification curve was observed but the curve was not valid and had Cq values of 25.0 and 26.6 cycles, respectively. In the third case, in which a p.Gly719Ala variant was detected, an amplification curve was observed but variants were not formally identified because the amount of amplifiable DNA present in the cartridge (*EGFR* total Cq was 25.7 cycles) was low. In this third case, the sample-in comprised 5% neoplastic cells, which was also below the study testing requirements (i.e., at least 10% neoplastic cells).

In the remaining two cases, which included one Ex19del (deletion 24) and one Ex20ins (InsG), no amplification curve was observed with the Idylla™ EGFR Mutation Test; total Cq values (27.6 and 25.7 cycles, respectively) confirmed that low amounts of amplifiable DNA were present in the cartridges. The deletion 24 had a COSMIC database prevalence of 0.02%. Overall, the 10 discordant negative cases had a mean *EGFR* total Cq value of 24.0 ± 3.9.

#### Primary concordant but secondary discordant cases

The five cases that were primary concordant but secondary discordant included detection of three Ex19del and two p.Leu858Arg primary variants (Figure 2; Supplementary Table S3). In terms of sample types, three were progression samples and one was a baseline sample; for the remaining sample, this information was unknown.

AF in three of the five cases were unknown. Regarding these three cases, curve investigation of the Idylla™ EGFR Mutation Test runs indicated it was likely that the amount of amplifiable DNA present in the cartridge was too low for detection of p.Thr790Met. One sample had an AF of 4% and one sample had an estimated AF of 10%. Concerning the latter, third method analysis with ddPCR, using a residual DNA extract, was invalid due to the low amount of genomic DNA. The Cq total determined using the Idylla™ EGFR Mutation Test was relatively high (26.1 cycles) and may have been due to low quantities and/or poor quality of DNA. The five primary concordant but secondary discordant cases had a mean total *EGFR* Cq value of 23.0 ± 2.3 cycles.

#### Discordant-by-design cases

Thirteen cases of discordance by design occurred across seven different sites; 10 used amplicon-based NGS as the reference method, two used Sanger sequencing and one used hybrid capture NGS (Figure 2; Supplementary Table S3). Five of 13 variants were described in the COSMIC database and occurred as the following base changes (variant; prevalence in NSCLC, %): c.2127_2129del (p.Glu709_Thr710delinsAsp; 0.35%), c.2125G>A (p.Glu709Lys; 0.12%), c.2311_2319dup (p.Asn771_His773dup; 0.24%), c.2224G>A (p.Val742Ile; 0.03%) and c.2239_2240delinsCC (p.Leu747Pro; 0.29%). All 13 cases had a ‘no variant’ result with the Idylla™ EGFR Mutation Test with a mean (±SD) *EGFR* total Cq value of 23.2 ± 3.5 cycles.

#### Analytical turnaround time

**FIGURE 3:**
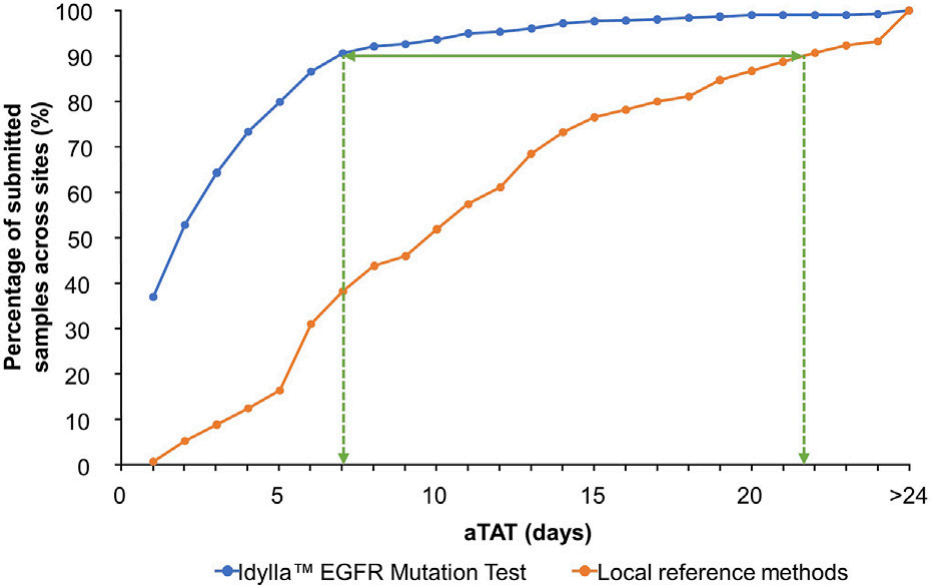
Overall cumulative percentage of tested sample results returned to the submitting clinician per aTAT: Idylla™ EGFR Mutation Test versus local reference methods. Percentage of submitted samples for which clinically actionable results were available per increasing aTAT when analyzed using the Idylla™ EGFR Mutation Test or reference methods across sites. aTAT, analytical turnaround time; *EGFR*, epidermal growth factor receptor.

At the time of data collection, seven sites had not completed aTAT analyses or did not record any aTAT data for the Idylla™ EGFR Mutation Test. Therefore, aTAT data were available for nine of 16 sites. With the Idylla™ EGFR Mutation Test, 90% (*n* = 810) of a total of 900 samples with recorded aTAT data were tested within 7 days. Whereas with the reference methods, samples with recorded aTAT data (*n* = 865) were tested within approximately 22 days (Figure 3; cumulative percentage of test results obtained plotted against aTAT). One site had a median aTAT of results with external NGS reference methods of 21.0 days (Site 13), which was much longer than the median aTAT of results for other sites. When excluding this site, the longest median aTAT of results using reference methods was 13.0 days (Site 5), which is in-line with previous reports [44]. The differences between aTAT values were calculated as single points (*n* = 865 for the Idylla™ EGFR Mutation Test aTAT and reference methods aTAT, and *n* = 580 for the Idylla™ EGFR Mutation Test aTAT and NGS reference methods only aTAT), to determine overall reduction in median aTAT with the Idylla™ EGFR Mutation Test and reference methods. Across the nine of 16 sites with available aTAT data, the Idylla™ EGFR Mutation Test reduced the overall median (Q1–Q3) aTAT of results by 7.0 days (4.0–13.0 days) compared with the aTAT of results from reference methods (*n* = 862), and by a median of 9.6 days (5.0–14.0 days) compared with the aTAT of results from NGS reference methods only (*n* = 580).

**FIGURE 4:**
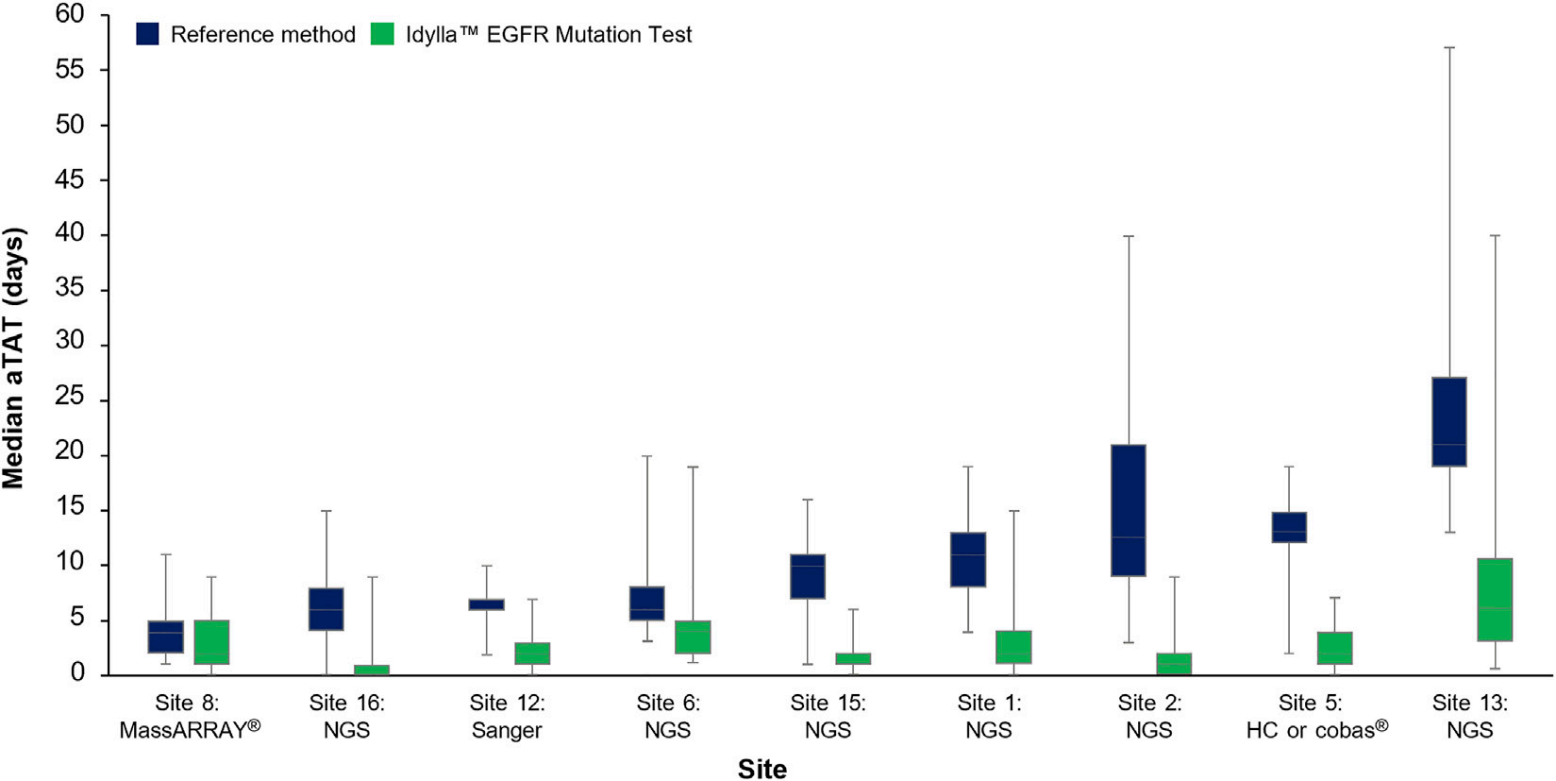
Median aTAT determined for Idylla™ and reference methods across sites where data were available. Data for each reference method (HC-NGS and cobas®) at Site 5 were assessed as one data set (involving a total of *n* = 97 samples) due to low sample numbers for each method if analyzed individually. Error bars represent minimum and maximum values; box plot represents Q1, median, and Q3. aTAT, analytical turnaround time; *EGFR*, epidermal growth factor receptor; HC, hybrid capture; NGS, next-generation sequencing.

The median (Q1–Q3) aTAT for Idylla™ EGFR Mutation Test results ranged from 1.0 day (0.0–1.0 day) to 6.0 days (3.0–10.5 days) across sites. The median (Q1–Q3) aTAT for reference methods results ranged from 4.0 days (2.0–5.0; MassARRAY®) to 21.0 days (18.8–26.0; outsourced NGS) across sites (Figure 4). Median (Q1–Q3) difference in aTAT between reference method and Idylla™ EGFR Mutation Test results varied across sites and, therefore, by the specific reference method used, ranging from 1.0 day (0.0–2.0; MassARRAY®) to 14.0 days (13.0–16.0; outsourced NGS; Table 2). In depth country-specific differences were not analyzed.

## Discussion

Clinical guidelines in NSCLC generally require that *EGFR* variant status is analyzed before a treatment plan is initiated to identify patients who are suitable for targeted treatments. *EGFR* variant testing should be conducted for common EGFR TKI-sensitizing variants (e.g., Ex19del or p.Leu858Arg in exon 21) in eligible patients with metastatic NSCLC and for patients with resectable stages IB to IIIA NSCLC [4,13,19,20,21,38]. It should also be used for identifying resistance variants in patients who relapse while receiving first or second-generation EGFR-TKIs [4]. Additionally, *EGFR* testing is recommended for rarer *EGFR* variants (e.g., *EGFR* Ex20ins variants; *EGFR* p.Ser768Ile, p.Leu861Gln and/or p.Gly719X) in eligible patients with metastatic NSCLC [13].

Various methods are used in routine *EGFR* variant testing; however, most require specialized testing facilities, or external access to such facilities, with trained staff to perform analyses and interpret results [30]. These methods, particularly when outsourced, are often used to batch test patient samples [30]. The frequent requirement for batch testing, multiple preparatory procedures and results interpretation as well as potential transport delays, if testing is outsourced, can result in lengthy aTATs. The automated Idylla™ EGFR Mutation Test avoids need for specialized testing facilities, does not require trained staff, and can be operated rapidly, with a run time of approximately 150 min, on a targeted (non-batch-related) patient-by-patient basis [43, 45]. Performance of the assay has been reported in previous studies [39–41,46]. Incorporation of this *EGFR* variant test into existing clinical workflows is expected to help reduce aTAT and, in turn, reduce time to treatment initiation, especially where samples test positive for targetable *EGFR* variants. These rapid and accessible test types may be of high value in streamlining clinical workflows, both in every-day clinical practice and during national health crises, such as the COVID-19 pandemic. In fact, a recent study demonstrated that preference for PCR-based methodologies over highly multiplexing assay approaches was one of several local strategies implemented to help overcome COVID-19 pandemic-related delays and other related challenges in lung cancer clinical practice [47]. It should be noted that the FACILITATE study was not impacted by the COVID-19 pandemic due to timing of the study.

FACILITATE was a real-world, prospective, multicenter European study that evaluated performance and aTAT of the Idylla™ EGFR Mutation Test compared with local reference methods in real-life NSCLC settings. Across FACILITATE, there was a high level of concordance between the Idylla™ EGFR Mutation Test and local reference methods. No difference in OPA was observed for the Idylla™ EGFR Mutation Test versus all reference methods, or Idylla™ EGFR Mutation Test versus NGS reference methods only. The high level of concordance illustrates robustness and accuracy of the Idylla™ EGFR Mutation Test in a real-world setting. Concordance was higher when discordance-by-design cases were excluded. Cases classified as discordant by design involved specific *EGFR* variants that were detected by the reference method but not by the Idylla™ EGFR Mutation Test because those specific variants were not part of the Idylla™ 51-member panel.

Regarding discordant cases, these represented less than 2% of all analyses across the study when excluding for discordance-by-design cases. Most of these non-design-related discordances were resolved on third-method analysis; the main causes of discordance were insufficient material, low quality sample input and/or low AF. This can be explained because the Idylla™ EGFR Mutation Test does not allow for in-process quality control of extracted DNA, therefore, selection based on DNA quality was not possible. As a result, samples with low analyzable DNA content (indicated by high mean *EGFR* control Cq values) [48] or low numbers of mutated cells may have led to an increased risk of false-negative results. Furthermore, it is important to consider the effects of tumor heterogeneity on the discordance observed in this study. Single samples collected for tissue testing cannot truly encompass the diverse profile of the tumor due to inter-metastatic and even intra-tumor heterogeneity. The latter of which can also contribute to false results [49–51].

Five cases (three confirmed progression samples; one confirmed baseline sample; one was unknown as to if it was a progression or baseline sample) were concordant for a primary variant but discordant for *EGFR* p.Thr790Met. The relatively high frequency at which *EGFR* p.Thr790Met discordances occurred was expected, because the Idylla™ EGFR Mutation Test versus reference methods has lower inherent technical performance in detecting *EGFR* p.Thr790Met resistance variants compared with primary variants. The sensitivity of the Idylla™ EGFR Mutation Test for p.Thr790Met is design-limited to an allelic frequency of ≥ 5%; this design limitation is in place to avoid risk of false-positive results caused by deamination-related issues that can affect p.Thr790Met [45]. It should be noted that the one confirmed baseline sample in which the p.Thr790Met variant was detectable with the local reference method but not with the Idylla™ EGFR Mutation Test, was positive for primary variants with both methods. In view of this and because osimertinib is currently the preferred first-line treatment in EGFR-TKI-sensitizing variant-positive (eg, Ex19del, p.Leu858Arg) advanced NSCLC [4, 13, 19], cases of primary variant concordance but p.Thr790Met variant discordance are of less clinical importance at diagnosis. This is because osimertinib targets p.Thr790Met variants irrespective of whether they are primary or acquired [14]. However, in cases of NSCLC progression on first- or second-generation EGFR-TKIs, reliable and accurate p.Thr790Met variant testing remains essential [4].

In terms of discordant-by-design cases, the Idylla™ EGFR Mutation test is designed to test primarily for commonly occurring NSCLC-associated *EGFR* primary variants, including the group of Ex19del variants and the p.Leu858Arg variant in exon 21 [6], with rapid aTAT of results. Although, the 51 *EGFR* variants included in the Idylla™ EGFR Mutation test panel [43] does account for several rarer (“uncommon”) clinically relevant variants [such as p.Gly719Ala, p.Gly719Ser and p.Gly719Cys in exon 18, p.Ser768Ile in exon 20, five Ex20ins (p.Asp770_Asn771insGly, p.Val769_Asp770insAlaSerVal, p.Val769_Asp770insAlaSerVal, p.Asp770_Asn771insSerValAsp and p.His773_Val774insHis), and p.Leu861Gln in exon 21] [43], other uncommon *EGFR* variants are not included in the Idylla™ EGFR Mutation test panel, and therefore are undetectable (by design) with the Idylla™ EGFR Mutation test. More comprehensive techniques can be used to detect whether these variants are present. In FACILITATE, less than 1% of samples were designated discordant by design. Although rare, these *EGFR* variants should be accounted for in clinical practice, highlighting the recommendation for complementary testing of negative samples (by the Idylla™ EGFR Mutation Test) by NGS. This integrated workflow could ensure that any uncommon *EGFR* variants undetectable by the Idylla™ EGFR Mutation Test are captured by NGS [52, 53]. However, while it would be preferable for the Idylla™ EGFR Mutation Test panel to include all these variants, there are likely to be few implications of these remaining undetected, considering approved treatments are lacking for most of these missing, uncommon variants [54].

Preliminary clinical data suggest that some uncommon variants are sensitive to EGFR-TKI treatment, as noted in a recent review [55]; however, clinical data interpretation may be confounded by the concomitant presence of complex variants. Moreover, reports regarding uncommon variants in NSCLC can be limited by small patient populations and the often-retrospective nature of these studies, which makes these data hard to interpret. As such, more clinical studies in patients with uncommon, rare, and complex *EGFR* variants are needed. It should be noted that increasing numbers of new treatments are being developed for targeting rare variants including those related to exon 20 [56] and, therefore, the amount of published clinical data regarding such rare variants is expected to increase.

The total percentage of discordant results reported in FACILITATE may be lower than expected in clinical practice. This is because there was an unanticipated bias by clinicians in the study to submit suitably or generously sized patient samples with sufficient neoplastic cells and exclude smaller, more challenging samples (i.e., patient samples with suboptimal quantities of material available for genetic testing using both the Idylla™ EGFR Mutation Test and the respective local reference method). This is likely due to the risk and clinical consequences of obtaining no or invalid results for patients for whom sample quantities were limited. The result of this understandable bias may mean that sample quantity-related issues related to the Idylla™ EGFR Mutation Test and the reference methods are underrepresented to some extent.

In our study, the Idylla™ EGFR Mutation Test reduced overall aTAT of results by a median of 1 week versus local reference methods results. Moreover, per cumulative analysis of samples processed per aTAT, results were available for 90% of samples within 1 week versus approximately 3 weeks using the Idylla™ EGFR Mutation Test versus local reference methods, respectively. The overall results indicate that incorporation of the Idylla™ EGFR Mutation Test into clinical workflows may be advantageous. The assay detects the most common clinically relevant *EGFR* variants and, therefore, may serve as a rapid and targeted sample-to-result screening technology conducted in parallel with more comprehensive variant testing (associated with longer aTAT of results). A recent report on data from 1,157 patients with advanced NSCLC in the UK indicated that approximately 25% of patients in whom targetable *EGFR* variants were reported did not receive targeted therapy initially [27]; one potential explanation noted was lengthy *EGFR* variant test turnaround times [27]. Incorporation of the Idylla™ EGFR Mutation Test into NSCLC clinical work flows thus may help mitigate clinician and patient delay-related motivations to initiate potentially suboptimal treatments while awaiting molecular testing results regarding *EGFR* variants [28, 31]. This may be particularly important in frail patients, patients with significant comorbidities or high symptom burden or patients with advanced disease including those with CNS metastases, all of whom are vulnerable to potentially rapid deterioration [28, 32, 33].

Most academic sites in our study used targeted NGS as their reference testing method that was conducted onsite; in community and private sites, specific testing methods varied and were sometimes outsourced. aTAT of results varied between sites and was typically shorter in academic sites than in community and private sites. This was expected as outsourcing is usually more common in smaller less equipped centers [57]. No country-specific differences could be deduced from the results as the study was not designed for this purpose. However, it can be concluded that in this study, aTAT of results was influenced predominantly by the methodology used at each site (Idylla™ EGFR Mutation Test vs. reference methods) rather than the country in which testing was performed. However, it should be noted that in standard clinical practice, country-specific reimbursement regulations may affect a center’s diagnostic testing strategy and, therefore, influence aTAT of results. Rapid single-gene testing (screening) completed in parallel with, or immediately followed by, comprehensive NGS testing is often the clinician’s preferred strategy, but this is not always feasible due to reimbursement restrictions.

In academic sites with comprehensive onsite specialized testing facilities, incorporation of the Idylla™ EGFR Mutation test may be importantly useful for designing fast-track workflows for triaging and quickly identifying patients eligible for EGFR-TKI treatment. In community or private sites where testing is often conducted offsite, aside from also helping with effective workflow design, the Idylla™ EGFR Mutation Test could importantly permit rapid onsite single-gene profiling. This may be critical given the risk of offsite batch testing- and sample transport-related delays, and the associated clinical implications for patients who are later found to be positive for common and actionable *EGFR* variants. However, it should be kept in mind that per ESMO precision working group recommendations, multiparametric NGS approaches should be favored from the outset and conducted as soon as possible to test for the presence of actionable variants [23]. In this way, patients who are found to be negative for common clinically relevant *EGFR* variants with the more rapid Idylla™ EGFR Mutation Test, will not experience added delays in obtaining comprehensive gene profiling results.

Irrespective of site-type and location, if the results using the Idylla™ EGFR Mutation Test are positive, appropriate targeted treatments could be started sooner, while comprehensive testing continues in the background. Currently, osimertinib is an applicable adjuvant targeted therapy in patients with resectable NSCLC, provided the patient’s disease has an appropriate molecular profile [23]. Therefore, an accurate, rapid, and *EGFR*-specific variant test such as the Idylla™ EGFR Mutation Test could be a beneficial and cost-effective approach in facilitating treatment decisions in patients with resectable early-stage NSCLC. It should be re-emphasized that rare or co-occurring variants will not be detected with this test. As such, in any patient with NSCLC, when results are negative with the Idylla™ EGFR Mutation Test (i.e., no common and clinically relevant *EGFR* variants identified) more comprehensive molecular testing results are required as soon as possible. Therefore, as noted previously, comprehensive testing should be conducted from the outset in parallel with this more rapid *EGFR*-specific approach, if used [23]. This is particularly important in advanced NSCLC because targeted treatments are available for several molecular targets (not limited to EGFR). Related to this and in all scenarios, clinicians should ensure that sufficient tissue sample quantity is always available for comprehensive molecular testing. If there is limited tissue available, use of tissue samples for comprehensive molecular testing should be prioritized.

Limitations of this study include an unanticipated clinician-driven sample-size selection bias that may have meant that the rate of discordances and invalid or non-processable results were underrepresented compared with that expected in clinical practice. Also, although the overall data were analyzable, data were not sufficient to permit site-specific aTAT determination at some sites or an analysis of the results by patient demographics. This was expected as FACILITATE was conducted in a real-world setting, in which incomplete record keeping and missing data are common [58]. Some clinicians may not have had the opportunity to record the exact time at which they submitted samples and/or received results. This was made more difficult by the high sample numbers analyzed at each site, each of which had only one Idylla™ console available for *EGFR* analysis, meaning that test results were not always returned at convenient hours when the submitting clinician was practicing, and therefore, other clinicians may have received the results on their behalf. In these instances, record keeping may have been less detailed. Lastly, the FACILITATE study did not analyze data on the impact of turnaround time on date of targeted therapy initiation where indicated. However, a recent bicentric prospective study [54] showed that aTAT of results (defined in that study as time from tumor sampling to initiation of EGFR-TKIs) was reduced by 12.5 calendar days when using the Idylla™ EGFR Mutation Test versus NGS with a reported OPA of 96.4%. The results of that bicentric prospective study [54], therefore, support the results of FACILITATE (a comparatively larger and multicentric study) concerning overall median aTAT reduction (7.0 days; from time of sample receipt by laboratory to when results were ready to be sent to the clinician) when using the Idylla™ EGFR Mutation Test versus local reference methods (NGS and several other methods).

## Conclusion and outlook

We demonstrate in a real-world multicenter setting, using samples with equal to or more than 10% neoplastic cell content, that the Idylla™ EGFR Mutation Test performed very well (∼98% OPA) compared with routinely used *EGFR* variant reference tests. Using an analysis of cumulative percentage of tested sample results returned by aTAT, we also show that the Idylla™ EGFR Mutation Test was able to deliver actionable and clinically relevant results typically in less time compared with local reference methods. The Idylla™ EGFR Mutation Test may serve as an enabling technology to detect common clinically relevant *EGFR* variants in NSCLC, either to complement existing workflows permitting a fast-track route, or to establish in-house *EGFR*-specific initial testing in sites without in-house testing facilities. However, in all cases, the feasibility of comprehensive molecular testing should be considered especially in cases where limited quantities of tissue sample are available; this is important given the possibility that common clinically relevant *EGFR* variants are not present. Considering the availability of targeted treatments such as EGFR-TKIs, the Idylla™ EGFR Mutation Test is a feasible and convenient means to help facilitate the application of personalized medicine in NSCLC.

## Data Availability

The raw data supporting the conclusion of this article will be made available by the authors, without undue reservation.

## Abbreviations

AF, allele frequency; aTAT, analytical turnaround time; CI, confidence interval; CNS, central nervous system; Cq, quantitation cycle; EGFR, epidermal growth factor receptor; ddPCR, digital droplet polymerase chain reaction; ESMO, European Society for Medical Oncology; Ex19del, exon 19 deletions; Ex20ins, exon 20 insertions; FFPE, formalin-fixed and paraffin-embedded; HC, hybrid capture; ND, no data; NGS, next-generation sequencing; NPA, negative percentage diagnostic agreement; NSCLC, non-small cell lung cancer; OPA, overall percentage diagnostic agreement; PCR, polymerase chain reaction; PPA, positive percentage diagnostic agreement; Q, quartile; RT-PCR, real-time polymerase chain reaction; SD, standard deviation; TKI, tyrosine kinase inhibitor.
