# Supplementary material for: FACILITATE: A real-world, multicenter, prospective study investigating the utility of a rapid, fully automated real-time PCR assay versus local reference methods for detecting epidermal growth factor receptor variants in NSCLC
Source: Pathol Oncol Res. 2023 Jan 31;29:1610707. doi: 10.3389/pore.2023.1610707 (PMC9927408; doi:10.3389/pore.2023.1610707)
Supplement: Supplementary file 1 [file Table1.DOCX]

**Supplementary Table S1 │** EGFR variants detectable using the Idylla™ EGFR Mutation Test.

| **Gene** | **Colloquial variant group nomenclature** | **Specific variant nomenclature** | **Base change/alteration** |
| --- | --- | --- | --- |
| Exon 18 | Gly719A | p.Gly719Ala | c.2156G>C |
|  | Gly719C | p.Gly719Cys | c.2155G>T  c.2154_2155delinsTT |
|  | Gly719S | p.Gly719Ser | c.2155G>A |
| Exon 19 | Del9 | p.Leu747_Ala750delinsPro | c.2238_2248delinsGC |
|  |  |  | c.2239_2248delinsC |
|  |  | p.Leu747_Ala750delinsSer | c.2240_2248del |
|  |  | p.Leu747_Glu749del | c.2239_2247del |
|  | Del12 | p.Leu747_Thr751delinsPro | c.2239_2251delinsC |
|  |  | p.Leu747_Thr751delinsSer | c.2240_2251del |
|  | Del15 | p.Glu746_Ala750del | c.2235_2249del |
|  |  |  | c.2236_2250del |
|  |  | p.Leu747_Thr751del | c.2239_2253del |
|  |  |  | c.2240_2254del |
|  |  |  | c.2238_2252del |
|  |  | p.Glu746_Thr751delinsAla | c.2237_2251del |
|  |  | p.Glu746_Thr751delinsIle | c.2235_2252delinsAAT |
|  |  | p.Glu746_Thr751delinsVal | c.2237_2252delinsT |
|  |  | p.Lys745_Ala750delinsThr | c.2234_2248del |
|  |  | p.Glu746_Thr751delinsLeu | c.2236_2253delinsCTA |
|  |  | p.Glu746_Thr751delinsVal | c.2237_2253delinsTA |
|  |  | p.Glu746_Thr751delinsAla | c.2235_2251delinsAG |
|  |  | p.Glu746_Thr751delinsGln | c.2236_2253delinsCAA |
|  |  | p.Ile744_Ala750delinsValLys | c.2230_2249delinsGTCAA |
|  | Del18 | p.Leu747_Pro753delinsSer | c.2240_2257del |
|  |  | p.Glu746_Ser752delinsVal | c.2237_2255delinsT |
|  |  | p.Leu747_Ser752del | c.2239_2256del |
|  |  | p.Glu746_Thr751del | c.2236_2253del |
|  |  | p.Leu747_Pro753delinsGln | c.2239_2258delinsCA |
|  |  | p.Glu746_Ser752delinsAla | c.2237_2254del |
|  |  | p.Glu746_Ser752delinsAsp | c.2238_2255del |
|  |  | p.Glu746_P753delinsValSer | c.2237_2257delinsTCT |
|  |  | p.Glu746_Ser752delinsIle | c.2236_2255delinsAT |
|  |  | p.Glu746_Ser752delinsIle | c.2236_2256delinsATC |
|  |  | p.Glu746_Ser752delinsVal | c.2237_2256delinsTT |
|  |  |  | c.2237_2256delinsTC |
|  |  |  | c.2235_2255delinsGGT |
|  | Del21 | p.Leu747_Pro753del | c.2238_2258del |
|  |  | p.Glu746_Ser752del | c.2236_2256del |
|  | Del24 | p.Ser752_Ile759del | c.2253_2276del |
| Exon 20 | T790M | p.Thr790Met | c.2369C>T |
|  | S768I | p.Ser768Ile | c.2303G>T |
|  | InsG | p.Asp770_Asn771insGly | c.2310_2311insGGT |
|  | InsASV9 | p.Val769_Asp770insAlaSerVal | c.2307_2308insGCCAGCGTG |
|  | InsASV11 | p.Val769_Asp770insAlaSerVal | c.2309_2310delinsCCAGCGTGGAT |
|  | InsSVD | p.Asp770_Asn771insSerValAsp | c.2311_2312insGCGTGGACA |
|  | InsH | p.His773_Val774insHis | c.2319_2320insCAC |
| Exon 21 | L858R | p.Leu858Arg | c.2573T>G |
|  |  |  | c.2573_2574delinsGT |
|  |  |  | c.2573_2574delinsGA |
|  | L861Q | p.Leu861Gln | c.2582T>A |

*Del, deletion; EGFR, epidermal growth factor receptor; ins, insertion.*
