# Supplementary material for: FACILITATE: A real-world, multicenter, prospective study investigating the utility of a rapid, fully automated real-time PCR assay versus local reference methods for detecting epidermal growth factor receptor variants in NSCLC
Source: Pathol Oncol Res. 2023 Jan 31;29:1610707. doi: 10.3389/pore.2023.1610707 (PMC9927408; doi:10.3389/pore.2023.1610707)
Supplement: Supplementary file 2 [file Table2.DOCX]

**Supplementary Table S2 │** Details of reference methods used per site.

| **Site number (arbitrary)** | **Site location** | **Reference method used** | **Manufacturer** | **Other details** |
| --- | --- | --- | --- | --- |
| 1 | Clermont-Ferrand, France | NGS | SOPHiA Genetics | Gene panel used: custom STS (45 genes) |
| 2 | Lyon, France | NGS | 1. Thermo Fisher Scientific 2. Illumina | Gene panels used:   1. Oncomine Solid Tumor panel 2. Archer RNA |
| 3 | Rennes, France | NGS | Production of libraries by an amplification technology on Juno platform (Fluidigm) and sequencing on MiSeq (Illumina) | Gene panel used: laboratory-developed panel |
| 4 | Orleans, France | NGS | Thermo Fisher Scientific | Gene panel(s) used: colon and lung |
| 5 | Hamburg, Germany | HC NGS or cobas^®^ | 1. NEO New Oncology GmbH 2. Roche Diagnostics GmbH | 1. NEOselect v1.0 2. cobas^®^ EGFR Mutation Test v2 |
| 6 | Berlin, Germany | NGS | Thermo Fisher Scientific | Gene panel designed by the National Network Genomic Medicine (Lung cancer), nNGM v1.0 (custom panel) |
| 7 | Dessau, Germany | NGS | Illumina | MiniSeq Illumina |
| 8 | Udine, Italy | MassARRAY^®^ | Diatech pharmacogenetics | Gene panel(s) used: Myriapod^®^ Lung status |
| 9 | Naples, Italy | Entrogen RT-PCR | CFX | Entrogen EGFR Analysis Kit |
| 10 | Bergamo, Italy | Therascreen^®^ EGFR RGQ PCR | QIAGEN | 29 somatic mutation as described in the Therascreen^®^ EGFR RGQ PCR kit user’s manual |
| 11 | Rozanno, Italy | MassARRAY^®^ | Diatech pharmacogenetics | Gene panel(s) used: Myriapod® Lung status |
| 12 | Rome, Italy | Sanger sequencing | Laboratory developed |  |
| 13 | Liege, Belgium | NGS | Multiplicon, Agilent | Gene panel(s) used: Tumor Hotspot MASTR Plus |
| 14 | Bordeaux, France | NGS | Thermo Fisher Scientific, Roche | Gene panels used:  NGS: routine CHU Bordeaux: Torrent™ Ion S5 kit Ion Ampliseq (custom panel) |
| 15 | Frankfurt, Germany | NGS | QIAGEN | Gene panel(s) used: Qiaact DNA/UMI + Lung RNA Fusion UMI panel |
| 16 | Tübingen, Germany | NGS | Thermo Fisher Scientific | Gene panel used: AmpliSeq Custom Panel |

*HC, hybrid capture; EGFR, epidermal growth factor receptor; NGS, next-generation sequencing; RGQ, rotor gene Q; RNA, ribonucleic acid; RT-PCR, real-time polymerase chain reaction; STS, solid tumor solution.*
