# Supplementary material for: FACILITATE: A real-world, multicenter, prospective study investigating the utility of a rapid, fully automated real-time PCR assay versus local reference methods for detecting epidermal growth factor receptor variants in NSCLC
Source: Pathol Oncol Res. 2023 Jan 31;29:1610707. doi: 10.3389/pore.2023.1610707 (PMC9927408; doi:10.3389/pore.2023.1610707)
Supplement: Supplementary file 3 [file Table3.DOCX]

**Supplementary Table S3 │** In-depth discordance analysis

| **Site** | **Idylla™**  **result** | **Reference method** | **Reference**  **result** | **Cq (cycles)**  **Idylla™** | **Remark** | **Most reliable test per third method analysis** |
| --- | --- | --- | --- | --- | --- | --- |
| **Discordant positive** | | | | | | |
| 3 | Ex19del | NGS | No variant | 24.5 | Third method analysis confirmed no variant | Reference |
| 3 | Ex19del | NGS | No variant | 26.5 | Third method analysis (other NGS method and fragment length) confirmed no variant | Reference |
| 7 | Ex20ins | Pyrosequencing | No variant | 25.3 | Third method analysis* confirmed exon 20 insertion | Idylla™ |
| 7 | Ex19del | Pyrosequencing | No variant | 21.8 | Third method analysis* confirmed no variant | Reference |
| 9 | Ex19del | Entrogen | No variant | 25.5 | No third method analysis | NA |
| 12 | p.Leu858Arg | Sanger | No variant | 23.9 | Third method analysis* confirmed p.Leu858Arg | Idylla™ |
| 12 | Ex20ins | Sanger | No variant | 21.6 | Third method analysis* confirmed no variant | Reference |
| 12 | p.Leu858Arg | Sanger | No variant | 25.4 | Third method analysis* confirmed p.Leu858Arg | Idylla™ |
| 12 | p.Ser768Ile | Sanger | No variant | 23.6 | Third method analysis* confirmed no variant | Reference |
| 15 | p.Thr790Met | NGS | No variant | 23.9 | Third method (ddPCR) analysis performed on left-over material in cartridge; inconclusive results | NA |
| **Discordant negative** | | | | | | |
| 1 | No variant | NGS | p.Leu858Arg | 27.3 | No third method analysis;  AF of 3% (NGS); no amplification curve observed | NA |
| 6 | No variant | NGS | Exon 19: c.2253_2276del (p.Ser752_Ile759del) | 27.6 | No third method analysis;  AF of 40% (NGS); no amplification curve observed | NA |
| 9 | No variant | Entrogen | p.Leu861Gln | 25.0 | No third method analysis; amplification curve observed | NA |
| 9 | No variant | Entrogen | p.Leu858Arg | 26.6 | No third method analysis; amplification curve observed | NA |
| 9 | No variant | Entrogen | p.Leu861Gln | 21.6 | Third method (ddPCR) analysis performed on left-over material in cartridge; p.Leu861Gln observed with AF of 0.4% | NA |
| 9 | No variant | Entrogen | p.Gly719X + p.Thr790Met | 14.7 | Third method (ddPCR) analysis performed on left-over material in cartridge; no variant detected | Idylla™ |
| 9 | No variant | Entrogen | Exon 20: c.2310_2311insGGT (p.Asp770_Asn771insGly) | 25.7 | No third method analysis;  no amplification curve observed | NA |
| 13 | No variant | NGS | p.Gly719Ala | 25.7 | No third method analysis; amplification curve observed | NA |
| 14 | No variant | NGS | p.Leu861Gln | 24.6 | No third method analysis; AF of 4% (NGS); amplification curve observed | NA |
| 15 | No variant | NGS | p.Leu858Arg | 21.5 | H&E-stained sample used | NA |
| **Primary concordant, secondary p.Thr790Met discordant** | | | | | | |
| 3 | p.Leu858Arg | NGS | p.Leu858Arg + p.Thr790Met | 20.1 | Progression sample used; NGS in duplicate (routine protocol); AF of 4% | NA |
| 8 | p.Leu858Arg | MassARRAY^®^ | p.Leu858Arg + p.Thr790Met | 21.6 | Baseline sample used; no third method analysis; amplification curve observed; unknown AF | NA |
| 11 | Ex19del | MassARRAY^®^ | Exon 19 Deletion + p.Thr790Met | 24.2 | Progression sample used; no third method amplification curve observed; unknown AF | NA |
| 11 | Ex19del | MassARRAY^®^ | Exon 19 Deletion + p.Thr790Met | 22.8 | Progression sample used; no third method analysis; amplification curve observed; unknown AF | NA |
| 13 | Ex19del | NGS | Exon 19 Deletion + p.Thr790Met | 26.1 | Unknown if progression or baseline sample used; third method (ddPCR) analysis performed on residual DNA invalid due to low amount of gDNA; AF of 10% (NGS) | NA |
| **Discordant by design** | | | | | | |
| 2 | No variant | NGS | c.2127_2129del (p.Glu709_Thr710delinsAsp) | 21.8 | COSM51525  COMSIC%^†^: 0.35% | NA |
| 2 | No variant | NGS | c.2125G>A (p.Glu709Lys) | 22.3 | COSM12988  COSMIC%^†^: 0.12% | NA |
| 3 | No variant | NGS | c.2311_2319dup (p.Asn771_His773dup) | 19.2 | COSM12381  COSMIC%^†^: 0.24% | NA |
| 3 | No variant | NGS | c.2235_2248delinsAATTC (p.Glu746_Ala750delinsIlePro) | 27.2 | Not identified COSMIC database | NA |
| 5 | No variant | HC | c.1501G>A (p.Ala501Thr) | 23.6 | Not identified COSMIC database | NA |
| 6 | No variant | NGS | c.2310_2311insCCA | 24.4 | Not identified COSMIC database | NA |
| 12 | No variant | Sanger | c.2127_2129delAAC | 20.6 | Not identified COSMIC database | NA |
| 12 | No Variant | Sanger | c.2575G>A p.A859T | 27.3 | Not identified COSMIC database | NA |
| 13 | No Variant | NGS | c.2224G>A (p.Val742Ile) | 16.8 | COSM1090888  COSMIC%^†^: 0.03% | NA |
| 13 | No Variant | NGS | p.Leu692Val | 19 | Not identified COSMIC database | NA |
| 13 | No Variant | NGS | c.2138A>C (p.Lys713Thr) (+ KRASm) | 26.2 | Not identified COSMIC database | NA |
| 13 | No Variant | NGS | p.Gly779Phe | 27.3 | Not identified COSMIC database | NA |
| 14 | No Variant | NGS | c.2239_2240delinsCC (p.Leu747Pro) | 25.3 | COSM24267  COSMIC%^†^: 0.29% | NA |

‘Amplification curve observed’ indicates that the Idylla™ curve investigation found that there was a PCR amplification curve for the specific variant; however, the detection algorithm did not determine if the amplification signal represented a true variant signal. Potential root-causes of such amplification can include nonspecific amplification (background) or the presence of the variant but at an AF below the limit of detection of the Idylla™ Mutation Test at the given input level (ie, amount of amplifiable DNA in the cartridge reflected by the EGFR total Cq value).

*Alternative NGS method used as third method analysis.

^†^COSMIC% refers to the proportion of the respective EGFR mutation with respect to all NSCLC samples with EGFR mutations (9320) in the COSMIC database (data retrieval: 2022-01-13, <https://cancer.sanger.ac.uk/cosmic/browse/tissue>).

*AF, allelic frequency; COSMIC, Catalogue Of Somatic Mutations In Cancer; Cq, quantitation cycle; ddPCR, digital droplet polymerase chain reaction; DNA, deoxyribonucleic acid; EGFR, epidermal growth factor receptor; Ex19del, exon 19 deletion; Ex20ins, exon 20 insertion; gDNA, genomic DNA; H&E, haematoxylin and eosin staining; HC, hybrid capture; NA, not applicable; NGS, next-generation sequencing; NSCLC, non-small cell lung cancer; PCR, polymerase chain reaction; RNA, ribonucleic acid.*
